# A Prediction Error-driven Retrieval Procedure for Destabilizing and Rewriting Maladaptive Reward Memories in Hazardous Drinkers

**DOI:** 10.3791/56097

**Published:** 2018-01-05

**Authors:** Ravi K. Das, Grace Gale, Vanessa Hennessy, Sunjeev K. Kamboj

**Affiliations:** ^1^Clinical Psychopharmacology Unit, University College London

**Keywords:** Neuroscience, Issue 131, Reconsolidation, Memory, Prediction Error, Alcohol, Reward, Destabilization, Counterconditioning, Disgust, Plasticity

## Abstract

Maladaptive reward memories (MRMs) can become unstable following retrieval under certain conditions, allowing their modification by subsequent new learning. However, robust (well-rehearsed) and chronologically old MRMs, such as those underlying substance use disorders, do not destabilize easily when retrieved. A key determinate of memory destabilization during retrieval is prediction error (PE). We describe a retrieval procedure for alcohol MRMs in hazardous drinkers that specifically aims to maximize the generation of PE and therefore the likelihood of MRM destabilization. The procedure requires explicitly generating the expectancy of alcohol consumption and then violating this expectancy (withholding alcohol) following the presentation of a brief set of prototypical alcohol cue images (retrieval + PE). Control procedures involve presenting the same cue images, but allow alcohol to be consumed, generating minimal PE (retrieval-no PE) or generate PE without retrieval of alcohol MRMs, by presenting orange juice cues (no retrieval + PE). Subsequently, we describe a multisensory disgust-based counterconditioning procedure to probe MRM destabilization by re-writing alcohol cue-reward associations prior to reconsolidation. This procedure pairs alcohol cues with images invoking pathogen disgust and an extremely bitter-tasting solution (denatonium benzoate), generating gustatory disgust. Following retrieval + PE, but not no retrieval + PE or retrieval-no PE, counterconditioning produces evidence of MRM rewriting as indexed by lasting reductions in alcohol cue valuation, attentional capture, and alcohol craving.

**Figure:**
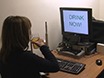


## Introduction

Building on seminal work of Lewis and colleagues^1^ over the last twenty years has highlighted an unprecedented potential for plasticity in established (consolidated) memories via the process of memory reconsolidation^2^,^3^. Reconsolidation occurs under certain circumstances when memories are retrieved^4^ and consists of two temporally^5^ and molecularly^6^ dissociable stages: initial destabilization^7^ and subsequent restabilization^8^. Following retrieval-induced memory destabilization, memories enter an 'active', malleable state, in which they are susceptible to modification through incorporation of novel information^9^,^10^,^11^,^12^, or weakening through pharmacological interference with the molecular pathways required for restabilization^13^,^14^,^15^,^16^. This 'reconsolidation window,' the interval between destabilization and restabilization, lasts 1 to 6 h and represents a unique opportunity to directly interfere with the maladaptive reward memories (MRMs) that are believed to play a key role in the aetiology^17^, progression^18^, and maintenance^19^,^20^ of substance use disorders (SUDs) like alcohol use disorder.

MRMS are associations formed through Pavlovian mechanisms that link environmental stimuli or 'drug cues' to the availability, and intoxicating and rewarding effects of drugs^21^. MRMs imbue drug cues with enhanced value, salience^22^, and motivational properties such that these cues grab attention^23^, trigger craving and motivate drug seeking-and-using behaviour in people with SUDs. These MRMs are therefore thought to be responsible for triggering relapse when drug cues are encountered^24^. The primary challenge in the long-term clinical management of SUDs is therefore to reduce the ability of MRMs to promote drug-taking, which, by extension, should reduce the incidence of relapse^21^. The reconsolidation window offers an exciting opportunity to achieve this aim by updating MRMs while they are unstable to a form that is less likely to contribute to relapse.

Recent research has shown that this can be achieved using purely behavioral interventions^10^,^25^. These can produce profound reductions in craving and drug-related reward metrics in heroin addicts^26^, smokers^27^, and hazardous drinkers^28^. By giving a brief reminder of MRMs (typically brief exposure to drug cues) to destabilize them, and subsequently re-associating drug cues with lack of drug (extinction), or aversive outcomes (counterconditioning)^29^ while MRMs are unstable, the associations between drug cues and outcomes restabilize in an updated, more adaptive form. These rewritten MRMs no longer support relapse. The only difference between these and standard extinction or counterconditioning interventions is the retrieval and destabilization of MRMs - produced by brief exposure to drug cues - prior to the learning intervention. They are therefore typically referred to as 'retrieval-extinction' or 'retrieval-counterconditioning' interventions.

However, there is now a significant body of research showing inconsistent results with these interventions^30^,^31^,^32^,^33^. This may be partially explained by individual differences in the expression of plasticity-promoting proteins^34^. However, since retrieval is necessary but not sufficient^4^,^35^,^36^ for memory destabilization, there are also certain boundary conditions that determine whether a memory destabilizes or not. The extent to which these boundary conditions were operative likely also varied from study-to-study. Chief among these conditions are memory age (chronology)^37^ and strength (reinforcement history)^38^,^39^, duration of the reminder trial^5^, and the occurrence of mismatch^35^,^40^ between predicted and actual outcome during retrieval, known as prediction error (PE)^41^,^42^,^43^. These factors are inter-related, as PE is the central reward 'learning signal'^44^,^45^ and diminishes over the course of learning. PE encodes the difference between expected outcome encoded in memory and actual outcome, reflecting the inaccuracy of learned predictions. It is by minimizing the occurrence of PE that learning accrues. Old, strongly reinforced memories like MRMs - which have formed over many years - have extremely good predictive power for outcomes across different situations. As such, little PE will be generated when MRMs are retrieved under normal conditions, conferring these traces with destabilization-resistance.

Leveraging this knowledge, we developed a retrieval and PE-generation (Retrieval + PE) procedure for alcohol MRMs that aims to maximize the likelihood of these memories destabilizing by maximizing PE at retrieval. We have shown that subsequent counterconditioning causes comprehensive updating of alcohol MRMs in hazardous drinkers^28^,^46^. This manuscript describes the implementation of the Retrieval + PE procedure for destabilizing alcohol MRMs, along with a multisensory counterconditioning technique that rewrites these memories.

The Retrieval + PE procedure involves the use of verbal instructions to maximize participants' expectancy of alcohol reward, then preventing alcohol consumption at the last moment. Without knowledge of the exact learning history, this PE maximization putatively maximizes the likelihood of MRMs destabilization and provides a platform for testing the efficacy of post-destabilization behavioral interventions in improving indices of maladaptive reward memory. Here we describe how to implement this procedure with subsequent disgust-based counterconditioning, which we^28^ and others^29^ have shown to be an effective learning modality for updating MRMs. However, the Retrieval + PE procedure could also be used in combination with alternative post-retrieval drug interventions and many other forms of learning-based interventions^25^.

## Protocol

All procedures were approved by the University College London Research Ethics Committee and carried out in accordance with the Declaration of Helsinki. Obtain all necessary local approval prior to using this method.

### 1. Preparation of Gustatory Disgust Unconditioned Stimulus

1. Using a micropipette with 1 mL pipette, add 80 µL of denatonium benzoate solution (2.5% in water) to a mixing glass.
2. Using a measuring cylinder, carefully measure 120 mL of clean water into the mixing glass containing the denatonium benzoate solution. Stir well to mix. This produces 120.08 mL of 0.067% denatonium benzoate.
3. Evenly divide the 0.067% denatonium benzoate solution into 8 clean, identical opaque cups.
  1. Divide the solution into cups using high-accuracy electronic scales. First, place an empty cup onto the scales and tare the scales to zero. Slowly add 0.067% denatonium benzoate solution until the scale reads 15 g. Repeat this for all 8 cups. This will give an equal amount of solution in each cup.
  2. Alternatively, using a 5 mL syringe, add 15 mL of 0.067% denatonium benzoate solution to the plastic cups. This will give an equal amount of solution in each cup.
4. Prior to participant arrival, arrange the 8 cups of bitter drink behind a screen such that they are easily reached by the experimenter, but out of sight of the participant. NOTE: Consumption of the contents of each cup serves as a gustatory disgust unconditional stimulus (UCS) trial.

### 2. Preparation of Participants and Drinking Measures

1. Using a randomization command in a statistical package (*e.g.*, using the 'set.seed' and 'sample' functions in R^47^) or a suitable online random allocation tool (*e.g.*, random.org^48^, randomize participants to one of the following intervention groups: (1) Retrieval + PE (**RET + PE**) (2) Retrieval + No PE (**RET + No PE**) (3) No Retrieval + PE (**No RET + PE**) such that allocation to each group is equal.
2. Confirm hazardous drinking with the Alcohol Use Disorders Identification Test (AUDIT)^49^. Scores >10 were considered acceptable for this study.
3. Assess level of alcohol dependence via Structured Clinical Interview for the Diagnostic and Statistical Manual-5 (SCID)^50^ criteria. Consider <4 items coded as threshold level as acceptable (*i.e.* exclude those with current alcohol use disorder). Refer to section 9 for more information on inclusion and exclusion criteria. NOTE: If using the current stimulus set, participants must self-report primarily drinking beer (>60% total alcohol consumption). Exact inclusion/exclusion criteria will depend upon the nature and purpose of the study. Ensure participants are excluded if they are contraindicated for counterconditioning or exposure to alcohol cues.
4. Obtain informed consent from participants.
5. Use an alcohol breathalyzer testing device to ensure participants are not intoxicated. If the reading is above 0.001 mg/L, re-arrange testing day or exclude the participant.
6. Seat participants in front of the computer to be used to display stimuli.
7. Assess state (in-the moment) craving for alcohol at baseline and again after exposure to cues with the Alcohol Craving Questionnaire (ACQ-NOW)^51^ and alcohol consumption over the previous week at baseline and follow up (Day 8) using the Timeline Follow Back for Alcohol (TLFB)^52^ .
8. Use the Stages of Change Readiness and Treatment Eagerness Scale (SOCRATES)^53^ to determine drinking concern and readiness to change, the Disgust Propensity and Sensitivity Scale Revised (DPSS-R) to index propensity and sensitivity to disgust^54^, and the Negative Alcohol Expectancy Questionnaire (NAEQ) for negative drinking expectancies^55^.
9. Use stimulus presentation software to present all stimuli during the memory reactivation and counterconditioning procedure (Steps 3.2.2. to 3.3.4 and and 5.1. to 6.4.).

### 3. Memory Reactivation and Control Non-reactivation Procedures

NOTE: See Figure 1 for the schematic.

1. For participants in the RET + PE and RET-no PE groups, pour 150 mL of chilled alcohol-free beer into a half-pint (284 mL) glass and place this on the table between participants and the display screen. For those in the No RET + PE group, pour 150 mL of chilled orange juice into the glass and place this on the table in front of participants.
2. For stimulus rating, present instructions and stimuli for the relevant retrieval/no retrieval procedures. NOTE: The exact instructions used in a published study^26^ are available upon request from the authors.
  1. Tell participants that the glass in front of them contains beer or orange juice, as appropriate to their group. Inform them that they will consume this drink according to on-screen instructions after rating a number of pictures for pleasantness and effects on 'urge to drink' the beer or juice in front of them.
  2. Explain to participants that the 11-point pleasantness scale runs from -5 (extremely unpleasant) through 0 (neither pleasant nor unpleasant) to +5 (extremely pleasant). For the urge to drink scale, explain that it runs from -5 (greatly reduces urge to drink) through 0 (has no effect on urge) to +5 (greatly increases urge to drink). Instruct participants to make all ratings out loud.
  3. Tell participants to recall previous instances when they drank beer (or orange juice) to guide their ratings.
  4. Show participants examples of the on-screen instructions they will see when they are required to consume the drink in front of the, e.g., "PICK UP DRINK" in black text, "PREPARE TO DRINK" in blue text, and "DRINK NOW" in green text. Instruct the participants to only drink when they see 'DRINK NOW' written in green text.
  5. Deploy the reactivation/control task in the relevant manner for the stimulus presentation software being used. Program the task to present conditioned stimulus (CS) images for 10 s each in a pseudo-randomized order. This order was determined by options within the software used to program the task and stipulates no more than two consecutive presentations of Beer CS. Have the participants drink only when "DRINK NOW" in green text is presented.
  6. Record participants' pleasantness/urge ratings in response to four beer images (alcohol cues; CS+s) and two non-alcohol rewarding drink images (neutral cues CS-s*,i.e.* coffee and cola) for the RET+PE and RET-no PE groups, or to four orange juice images and two CS-s for the No RET + PE group.
3. ***In Vivo* Rating and Prediction Error Procedure**
  1. After all images have been rated, direct participants' attention to the drink in front of them. Ask them to rate (the sight of) the drink itself for pleasantness and how much it affects their current urge to drink (both on the same -5 to +5 scales previously used).
    1. Instruct participants to imagine consuming the drink and rate how pleasant they think they will find it from -5 (extremely unpleasant) to 5 (extremely pleasant).
    2. Instruct participants to imagine consuming the drink and rate their current urge to consume the drink from -5 (least I have ever wanted to drink) to +5 (most I have ever wanted to drink). Note the different wording to the previous -5 to +5 urge scale.
  2. Begin the on-screen drinking instructions.
    1. For all participants, present the first two screens that read "PICK UP DRINK" and "PREPARE TO DRINK", respectively. For those in RET + PE and No RET + PE groups, present the final screen that reads 'STOP! DO NOT DRINK!'. The participants must not drink the beer or orange juice, engendering negative prediction error.
    2. For those in RET-no PE group, present the final screen that reads "DRINK NOW" and have the participants consume the beer. Display all drinking-instruction screens for 5 s.
    3. After consuming or not consuming the drink, show the screen that reads 'put down the drink and press the space bar to continue'. Ensure compliance with this instruction.
  3. Remove the glass from the sight of the participants.
  4. Have the participants rate out loud how expected/unexpected the preceding instructions were from -5 (completely unexpected) to 5 (completely expected). Write down the response.

### 4. Distractor Tasks

1. Place headphones on participants.
2. Immediately perform the series of short term memory tests in the order listed below.
  1. First, play one of the prose recall versions from the Rivermead Behavioural Memory Test^56^. Elicit immediate recall by asking participants to write down as much detail about the story as they can.
  2. Administer the digit span task^57^, following standard protocols for this task.
  3. Administer the verbal and category fluency tasks^58^, following standard protocols for task completion (60 s to name as many exemplars as possible).

### 5. Instructions for Counterconditioning Task

1. Implement the counterconditioning task in the same stimulus presentation software as used in the retrieval/no retrieval procedure outlined in step 3, so that it can proceed from the retrieval/no retrieval procedure. NOTE: A pre-deployed version of this task that will run on most PCs most operating systems is available upon request from the authors.
2. Begin the instructions for the counterconditioning task.
  1. Inform participants that they will view a series of pictures that will be followed by different outcomes displayed on the screen, such as another picture or the words 'DRINK NOW'.
  2. Take the first UCS drink from behind the screen and place this in front of the participant. Inform the participant that whenever they see the words 'DRINK NOW', they must pick up the cup and drink all the liquid inside. Inform participants that the drinks may taste very bitter but are not harmful. NOTE: Participants must be unaware of the total number of drinks or number remaining throughout counterconditioning.
  3. Instruct participants that whenever they see the first (CS) image on each trial, they must rate how pleasant they find the image from -5 (extremely unpleasant) to +5 (extremely pleasant).
  4. Have the participants use dedicated keys on the keyboard to make their ratings. Use the keys [backslash], [1], [2], [3], [4], [5], [6], [7], [8], [9] and [0] or alternative consecutive keys with stickers placed over them that read [-5], [-4], [-3], [-2], [-1], [0], [+1], [+2], [+3], [+4] and [+5], respectively. To remind participants to make this rating, have the words 'rate pleasantness now' appear on screen when the first image (CS) is displayed.
  5. Inform participants that after the outcome has occurred (UCS drink or another picture) to rate the pleasantness of the outcome on the same -5 (extremely unpleasant) to +5 (extremely pleasant) scale. This refers to either the consumption of the bitter drink UCS or presentation of the pictorial UCS. NOTE: The next trial in the task does not begin until these ratings are made.
  6. Reiterate that the participants will therefore be making two ratings per trial, one for the initial picture (CS) and the second for the outcome (UCS).
  7. Ensure that participants understand these instructions and begin the counterconditioning task.

### 6. Running Counterconditioning Task

1. Use 4 beer stimuli as CS+s (the 4 beer stimuli used in the retrieval task) and two neutral stimuli as CS-s (the coffee and cola images used in the retrieval/no retrieval task). Prepare three UCS "Types". The first type: "Gustatory UCS" consists of the words "DRINK NOW" appearing on screen and the participants consuming the cup of bitter liquid placed in front of them. The second type: "Pictorial UCS", consists of presentation of one of the disgusting images detailed below. The third type: "Neutral UCS" consists of the neural images detailed below.
  1. Pair two of the beer CS+s (designated the "Drink CS+s") with the gustatory UCSs (these are labelled "gustatory" in the "UCS Type" column of **Table 1**). Following these CSs, let the words "DRINK NOW" appear on screen and ask participants to consume the cups of bitter liquid placed in front of them. Pair two (designated "Pictorial CS+s") with visual disgust-inducing UCSs from the International Affective Picture System (IAPS; three images: 9301.jpg, 9325.jpg, 9405.jpg, and a fourth sourced from the internet depicting a wound on a human foot that is infested with maggots; these CSs are labelled "Pictorial" in **Table 1**). NOTE: Allocation of beer CS to outcome should be random and made prior to beginning data collection. The allocation of CS to outcome type should then be kept constant across all participants. The trials and trial sequence are identical across all experimental groups. The outcome UCSs for the neutral CS-s are neutral-valence IAPS images (1020.jpg and 1021.jpg). All pairings are on a 100% reinforcement schedule.
  2. Pair each of the two pictorial CS+s with each visual disgust UCS image once (8 trials total). Pair each of the Gustatory CS+s with the words 'DRINK NOW' and consumption of the gustatory disgust UCSs (bitter drink) four times (8 trials total). Pair each of the neutral CS-s with the neutral UCS pictures four times (8 trials total). Therefore, perform a total of 24 trials.
2. Begin the counterconditioning task by pressing a dedicated key (spacebar) once the participant understands that they are required to make two ratings per trial: one for the initial picture (CS) and one for the outcome (UCS).
3. Use a pseudo-randomized trial order for all participants. A working example is given in in Table 1, with all 24 trials completed in a single sitting. NOTE: This order ensures that the same UCSs does not occur in consecutive trials and that no more than two trials of the same CS occur consecutively. However, an alternative trial ordering that fulfills these requirements may be used as long as it is consistent across participants.
  1. Have the participants in the **No RET + PE** group rate the four beer CS+s for pleasantness once, immediately prior to beginning counterconditioning. NOTE: This is to provide baseline rating in this group and to ensure that the volume of pre-exposure to the CSs to be counterconditioned is identical between groups. It is not necessary to do this in **RET + no PE** or **RET + PE**, as they have already rated the beer images.
  2. Take the first UCS drink from behind the screen and place this in front of the participant in preparation for the "DRINK NOW" instructions.
  3. Present the first (CS) image in the sequence from **Table 1** for 6 s. Have the participants rate how pleasant they find the image within the 6 s from -5 (extremely unpleasant) to +5 (extremely pleasant) when the first (CS) image on each trial is presented.
  4. Have the participants use dedicated keys on the keyboard to make their ratings. Use the keys [backslash], [1], [2], [3], [4], [5], [6], [7], [8], [9] and [0] or alternative consecutive keys with stickers placed over them that read [-5], [-4], [-3], [-2], [-1], [0], [+1], [+2], [+3], [+4] and [+5], respectively. To remind participants to make this rating, have the words 'rate pleasantness now' appear on screen when the first image (CS) is displayed.
  5. Present the "DRINK NOW" instructions for 6 s and have the participant pick up the cup and consume the liquid. Ensure the entire amount of the denatonium benzoate solution is consumed on each gustatory UCS counterconditioning trial. As soon as one drink is finished, remove the empty cup and replace with the next 15 mL cup of denatonium benzoate solution.
  6. Have the participants rate the pleasantness of the gustatory UCS outcome on the same -5 (extremely unpleasant) to +5 (extremely pleasant) scale used to rate the CS after they have consumed the entire 15 mL. NOTE: There is no time limit for the UCS ratings. Once the rating is made, a single trial is complete.
  7. Present the next CS in the sequence (a Neutral picture, CS-1 in **Table 1**) for 6 s. Have the participants rate how pleasant they find the image within this period using the response keys for -5 (extremely unpleasant) to +5 (extremely pleasant), as previously.
  8. Continue with presentation of the remaining 22 trials. NOTE: The entire counterconditioning task takes approximately 10 min.
4. Upon completion, have the participants consume two squares of milk chocolate to remove the residual bitter taste of the denatonium benzoate solution. Instruct them to allow the chocolate to melt on their tongue.

### 7. One-Week Follow Up Measures

1. Schedule this session for one week after the retrieval/counterconditioning procedure.
  1. Take an alcohol breathalyzer reading. If reading is above 0.001, re-arrange testing day or exclude participant. Record if a positive reading (>0.001 mg/L) is obtained.
2. Present all CSs used in the counterconditioning task plus three novel pictures of beer and three novel pictures of wine (10 alcohol images in total) for 10 s each in a pseudorandomized order. Have the participants rate pleasantness from -5 (extremely unpleasant) to +5 (extremely pleasant) and effects on urge to drink beer from -5 (greatly reduces urge) to + 5 (greatly increases urge) for every image. Record their responses on paper. NOTE: Novel picture ratings are taken to assess generalization of effects within the class of beer stimuli and to other types of alcohol.
3. Administer the ACQ-NOW^49^, alcohol TLFB^52^ for the previous week, DPSS-R^54^, and SOCRATES^53^. NOTE: The same follow-up measures can be be used repeatedly for additional, extended follow up.

### 8. Dot-probe Task (Optional)

1. If access to an eye-tracking set-up is available, complete an attentional bias dot-probe task using the images used in the counterconditioning task at this stage. NOTE: The details of this task are given in Das *et al.*^28^ and are not given here. This task is available upon request from the authors.

### 9. Inclusion and Exclusion Criteria

1. Use the following inclusion criteria for hazardous drinkers: Hazardous drinking (defined as a score >10 on the Alcohol Use Disorders Identification Test, but <4 items coded as 3 on the SCID), consumption of >3 units for females, >4 units for males on at least 4 days per week (8 g pure alcohol/unit), fluent English, and normal or corrected-to-normal color vision.
2. Use the following exclusion criteria: ages <18 and >65, past or current diagnosis of drug or alcohol use disorders, any currently medicated mental health issues, any current major physical health issue, current pregnancy, or breastfeeding. NOTE: Exact inclusion and exclusion criteria may vary depending upon the population under test using this procedure.

### 10. Data Pre-Processing and Analysis^59^

1. For the purposes of analyzing CS ratings, calculate four epochs for each CS Type during counterconditioning. For each participant, this is achieved by simply taking the average of each two consecutive trials of each type. For example, in the order given in **Table 1**, average the ratings of Trial 1 and Trial 3 to get the first epoch for Beer CS+1. NOTE: This will produce 4 "Trial" levels for each stimulus for the counterconditioning rating data. This is done to smooth out trial-to-trial variation in ratings that occur due to spurious order and expectancy effects.
2. Assess all data for normality, homogeneity of variance and sphericity (for repeated-measures with *k*>2 comparisons).
  1. Where homogeneity of variance is violated in one-way analysis of variance, use Welch's F test. Where sphericity is violated, use the Huynh-Feldt or Greenhouse Geisser correction, as appropriate to the level of epsilon^56^. Winsorize any outliers >3 s.d. away from the sample mean for that variable to a score 3 s.d. from the mean. Run analyses with and without this procedure to assess sensitivity of results to the procedure and report any divergence.
3. Assess whether there are any differences over counterconditioning in responding to the Gustatory CS+s and Pictorial CS+s. If not, collapse the two CS types for counterconditioning analysis. If there are differences, include CS type as an extra level in analyses of counterconditioning data.

## Representative Results

Compare group means at baseline for subjective measures. Groups should be equivalent on baseline intake of alcohol, disgust sensitivity/propensity, SOCRATES and NAEQ scores, and momentary craving (ACQ-NOW). If there are differences in these measures at baseline, analysis should continue using mixed-effects models and these measures should be included as random-effects^59^. Performance of these analyses are not described here. Assess frequency of positive alcohol breathalyzer readings in each group and report this as an outcome.

Pleasantness ratings of CS+s and CS-s did not differ at baseline (before counterconditioning), but diverged over trials of counterconditioning, with pleasantness ratings for beer CS+s reducing significantly through repeated pairing with the pictorial and bitter drink UCSs. If CS+s and CS-s differ at baseline, use a mixed-effects model with random intercepts and slopes to analyze these data. The CS-s are never paired with UCSs, so do not show a decline in pleasantness. This is represented by a CS Type (CS+ vs. CS-) x Trial (Baseline, Trial 1, 2, 3 & 4) interaction if counterconditioning has been successful, as indicated in Figure 2 (representative data from Das *et al.*^28^)

Evidence of rewriting of MRM networks is provided by a generalized reduction in pleasantness ratings to alcohol stimuli, but not neutral stimuli at follow up (Day 8). In the RET+PE group, reduced pleasantness ratings were evident in response to CS+s used in the retrieval/counterconditioning task (Beer CS+s), as well as to novel beer and wine images rated for the first time at follow up. Statistically this was evident in a CS Type (Beer CS+, Neutral CS-, Novel Beer, Novel Wine) x Group ( No RET+PE, RET+PE, RET no PE) interaction. Example results showing this pattern taken from Das *et al.*^28^ are shown in Figure 3.

Examine this pattern of results in the optional attentional bias task. Attentional bias is calculated as dwell time on a target alcohol image - dwell time on its composition-matched non-target pair image. A score of 0 indicates no bias, positive score attentional bias towards alcohol stimuli, and a negative score an oculomotor aversion to alcohol pictures. Representative results taken from Das *et al.*^28^ are shown in Figure 4. It can be seen that an oculomotor aversion to alcohol images at follow up (Day 8) was produced in the RET+PE group only, indicated by a significant Group x CS Type interaction.

**Figure:**
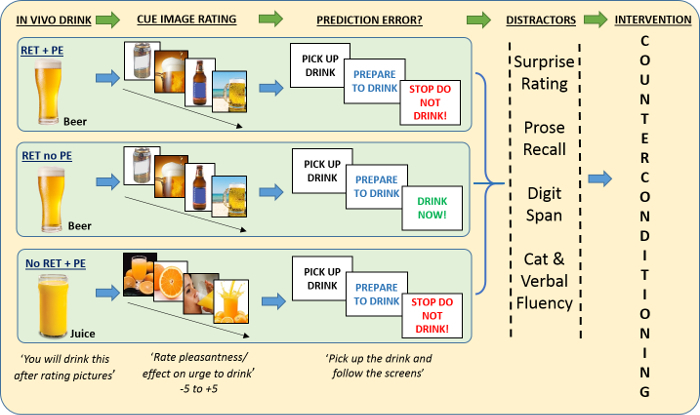

**Figure 1: A Schematic of the three retrieval groups and prediction error procedures.** Key to abbreviations: **RET + PE** = Retrieval + Prediction Error; the group who retrieve alcohol memories and have expected alcohol withheld. **RET + No PE** = Retrieval + No Prediction Error; the group who retrieve alcohol memories and consume alcohol as expected. **No RET + PE **= No retrieval + Prediction Error; this group does not retrieve alcohol memories and has expected orange juice withheld. ***In vivo***** drink**: A 150 mL glass of beer in RET + PE and RET + No PE or a 150 mL glass of orange juice in the no RET group. **1) *****In vivo***** drink:** The drink relevant to the particpants' group is placed in front of participants and they are told that they will consume this drink according to prompts they will see on screen; after rating a series of pictures for pleasantness. **2) Cue image rating:** Participants in RET + PE and RET + No PE rate four images of beer and two images of soft drinks (coffee and cola) for pleasantness. Brief *in vivo* exposure to beer and rating of beer images triggers retrieval of drinking memories in these groups. The No RET group rates four images of orange juice and the soft drink images for pleasantness and thus do not retrieve alcohol memories. **3) Prediction error:** As all participants are told that they will drink the *in vivo* drink at the start of the procedure, PE is engendered by preventing participants in RET + PE and No RET + PE from consuming the drink in front of them. In RET + No PE, the drink is consumed as expected, engendering no PE. **4)** **Distractors**: High working-memory tasks are performed (prose recall, digit span, and category and verbal fluency) to distract participants and disengage them from the retrieval task. **5)** **Intervention**: a counterconditioning task where beer images are paired with disgust-inducing images and very bitter drinks. This procedure aims to create an association between beer images and the experience of disgust that overwrites existing associations in the RET + PE group due to destabilization of alcohol memories. Please click here to view a larger version of this figure.

**Figure:**
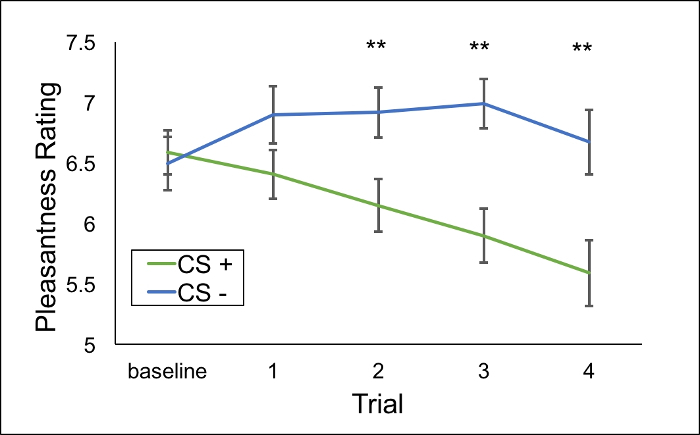

**Figure 2: Mean pleasantness ratings (± SEM) of neutral and beer cues during counterconditioning.** Representative changes in pleasantness ratings of CS+s (beer images paired with disgusting outcomes) and CS-s (neutral images paired with neutral outcomes) over the course of counterconditioning, taken from Das *et al.*^28^ As counterconditioning aims to invoke conditioned disgust in response to beer images, perceived pleasantness of these images should reduce during counterconditioning. Ratings do not differ at baseline, but reduce to CS+s over the course of counterconditioning. ** = CS+ > CS- at *p*< 0.001. Please click here to view a larger version of this figure.

**Figure:**
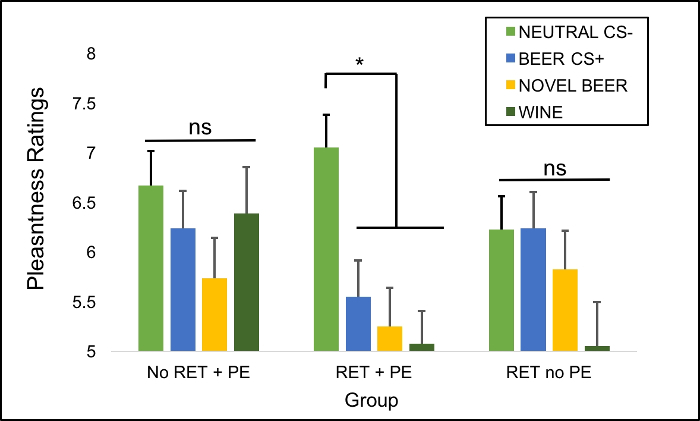

**Figure 3: Mean pleasantness ratings (± SEM) of counterconditioned, novel alcohol cues and neutral cues at test.** Representative generalized reductions in pleasantness ratings (liking) of alcohol images seven days after counterconditioning is performed following retrieval (RET) with prediction error (PE) (RET +PE) from* Das et al*.^28^ This pattern is consistent with rewriting of reward associations relating to alcohol in RET + PE. *Key: ***Neutral CS-** = coffee and cola images paired with neutral images during counterconditioning. **Beer CS+** = beer images paired with disgusting outcomes during counterconditioning. **Novel Beer** = previously unseen beer images. **Wine **= previousy unseen wine images. * = significant pairwise comparisons at *p*<0.05, Bonferroni corrected. Please click here to view a larger version of this figure.

**Figure:**
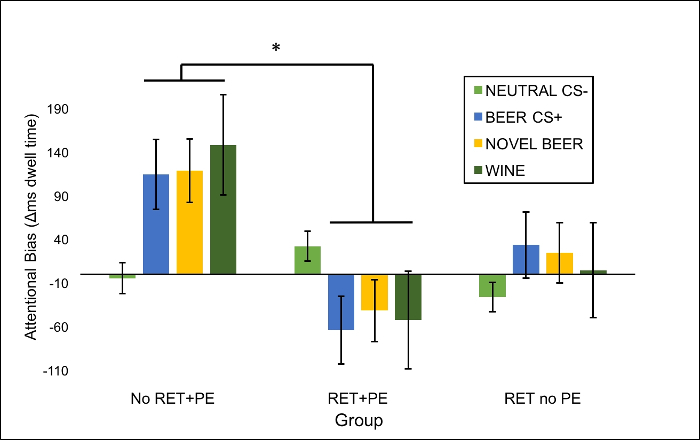

**Figure 4: Mean attentional bias to counterconditioned, novel alcohol cues and neutral cues at test.** Representative generalized abolition of attentional bias to all alcohol stimuli in the group that underwent counterconditioning after RET + PE from* Das et al*^28^. Bars indicate mean attentional bias scores, calculated as the averaged difference in total time looking at a 'target image' containing visible alcohol vs. a composition-matched 'non target' imagelacking visible alcohol. Error bars are SEMs.** Neutral CS-** = coffee and cola images paired with neutral images during counterconditioning. **Beer CS+** = beer images paired with disgusting outcomes during counterconditioning. **Novel Beer** = previously unseen beer images. **Wine **= previously unseen wine images. * = significant pairwise comparisons at *p*<0.05, Bonferroni corrected. Please click here to view a larger version of this figure.

**Table:** 

**TRIAL NUMBER**	**CS TYPE**	**UCS TYPE**	**UCS FILE NAME**
1	Beer CS+1	GUSTATORY	DRINK_SCREEN.PNG
2	Neutral CS-1	NEUTRAL	7020.jpg
3	Beer CS+1	GUSTATORY	DRINK_SCREEN.PNG
4	Neutral CS-2	NEUTRAL	7019.jpg
5	Beer CS+4	PICTORIAL	rotten_foot.jpg
6	Beer CS+3	PICTORIAL	9325.jpg
7	Beer CS+2	GUSTATORY	DRINK_SCREEN.PNG
8	Beer CS+4	PICTORIAL	9325.jpg
9	Beer CS+3	PICTORIAL	rotten_foot.jpg
10	Neutral CS-1	NEUTRAL	7020.jpg
11	Beer CS+1	GUSTATORY	DRINK_SCREEN.PNG
12	Neutral CS-2	NEUTRAL	7019.jpg
13	Neutral CS-1	NEUTRAL	7019.jpg
14	Beer CS+2	GUSTATORY	DRINK_SCREEN.PNG
15	Beer CS+4	PICTORIAL	9405.jpg
16	Beer CS+1	GUSTATORY	DRINK_SCREEN.PNG
17	Beer CS+4	PICTORIAL	9301.jpg
18	Neutral CS-2	NEUTRAL	7019.jpg
19	Beer CS+3	PICTORIAL	9405.jpg
20	Beer CS+2	GUSTATORY	DRINK_SCREEN.PNG
21	Beer CS+3	PICTORIAL	9301.jpg
22	Neutral CS-2	NEUTRAL	7019.jpg
23	Beer CS+2	GUSTATORY	DRINK_SCREEN.PNG
24	Neutral CS-1	NEUTRAL	7020.jpg

**Table 1: Pseudorandomized trial order for counterconditioning task used in Das *****et al*****.28 CS Type** = Conditioned stimulus type. There are four beer images used in the counterconditioning. These are arbitrarily numbered Beer CS+ 1 to 4. Two neutral images of coffee and cola are also used and these are arbitrarily numbered Neutral CS-1 and Neutral CS-2. **UCS Type** = Unconditioned Stimulus type. **Gustatory** unconditioned stimuli are the words "DRINK NOW" appearing on-screen, and participants consuming 15 mL 0.067% Denatonium Benzoate solution. **Pictorial** UCSs are unpleasant/disgusting images sourced from the International Affective Picture System (IAPS) database and internet. **Neutral **UCSs are two images sourced from the IAPS database that are rated neutrally. **UCS file name:** this column gives the specific images to be displayed as outcomes on each trial. Numeric file names refer to IAPS database numbers. These images are available upon request to the IAPS database. Other images are available from the authors on request.

## Discussion

The protocol in this paper describes an alcohol MRM retrieval procedure that explicitly generates prediction error in alcohol delivery (Retrieval + PE) to maximize the probability that naturalistic alcohol MRMs will destabilize. This retrieval procedure takes account of recent experimental work demonstrating the necessity of prediction error for memory destabilization and engaging the reconsolidation process^60^. It has been shown to produce the most profound changes in indices of alcohol MRMs from subsequent behavioral interventions (counterconditioning and cognitive reappraisal) in two papers^28^,^46^ and is currently being further validated in drug models. Since this effect appears to be independent of learning history, the procedure represents a significant improvement over other memory reactivation techniques, which do not incorporate a PE, and should be used as a platform for the assessment of post-destabilization interventions for reducing maladaptive drinking behaviour. Such refinement of techniques is particularly important in light of null findings in the reconsolidation field (see below). Moreover, the RET+PE method has considerable applied significance and may be applicable in combination with a variety of post-retrieval relearning methods. For example, we have demonstrated effects on semantic memory and craving using RET+PE followed by reappraisal, a prototypical adaptive emotion regulation technique commonly used in cognitive behavioural therapy^46^.

The procedure is easily modified to suit the characterisitics of the participant group. For example, the stimuli used as CS+s during counterconditioning can be changed to wine pictures, if the participants are primarily wine preferring. Alternatively, smoking-related stimuli can be used if the procedure is employed in tobacco smokers.

Effective counterconditioning relies on potent disgust cues. To ensure sufficient relearning during reconsolidation, select visual UCSs that are likely to elicit powerful disgust responses. Although these are visual cues, they should have relevance to gustatory disgust (*e.g.*, contamination-related images; images of food spoilage). If an alternative gustatory UCS (other than denatonium benzoate at the concentrations used here) is used, we recommend a compound that strongly activates bitterness responses.

Although we use an expectancy violation, it is theoretically possible to generate PE using other methods, although their efficacy in destabilizing robustly encoded reward memories has yet to be demonstrated. We have trialed an alternative procedure involving unexpected devaluation of alcohol (using unexpectedly bitter tasting alcohol), although we did not demonstrate convincing memory modifications using this procedure^46^.

Randomization of participants to groups should prevent baseline differences in trait questionnaire measures. If this is the case, however, these differences should be modelled as random effects in mixed-effects analyses of outcomes. A supplementary approach, which the authors recommend, is to include an extra baseline day prior to the retrieval and counterconditioning day. This allows better assessment of pre-existing group differences, the reliability of these differences, and gives researchers the option of stratified randomization to groups to prevent such differences.

In order to generate an effective PE, it is essential that participants experience a relevant surprising occurrence during retrieval. If an expectancy violation is used, as described here, ensure that participants are observed during this step so that they do not inadvertently consume the alcohol at a point they are instructed not to. Related to this, it is essential that participants are not aware of the experimenter's intention to withhold alcohol after generating the expectation of alcohol reward. As such, the protocol involves a necessary degree of deception. Upon debriefing, ensure participants are aware that they should not discuss the protocol with others.

While there is evidence that the procedure robustly destabilizes memories and has been designed to maximize the probability of doing so, there is currently no independent means of assessing whether or not destabilization has occurred. This is a limitation of all memory destabilization procedures described to date and as such, null effects of post-destabilization interventions at test are difficult to interpret, as they may be due to low efficacy of the intervention or a failure to sufficiently destabilize MRMs in the first place.

The remarkable generalization of Ret+PE dependent counterconditioning effects to non-trained stimuli makes this approach highly promising for clinical implementation. However, our demonstration of efficacy is limited to an experimental (non-clinical) setting with problem drinkers, who do not have an alcohol use disorder.

Relatedly, although the procedure is easily adapted, it is unknown whether the RET+PE procedure is similarly efficacious (in a modified form) in destabilizing MRMs in different (illicit) substance using populations. We believe it would be at least as effective, as alcohol MRMs are likely to be more overlearned, robust, and cross-contextual than MRMs for most illicit drugs. This remains to be verified experimentally however, and it is unknown how sensitive the efficacy of the procedure is to variations in number of cues presented or their exact nature. We therefore encourage experimentation with the procedure in different drug-using populations and with different retrieval cues, as well as tailoring post-destabilization interventions to population-specific reward maladaptations.

To address the measurement issues outlined above - namely the reliance on efficacy of the post-destabilization intervention to infer effective memory destabilization - independent measures of destabilization are required. We are currently developing electroencephalographic and psychophysiological techniques for resolving this issue and will make these available upon request when completed.

Implementation across the range of severities of problem drinking is required to establish the clinical utility of this technique. Moreover, clinically-oriented studies are essential to determine whether this procedure will be acceptable to treatment seekers.

## Disclosures

All authors have no intellectual, financial, or biomedical conflicts of interest to disclose.
